# Temperature-Dependent Photoluminescence Emission from Unstrained and Strained GaSe Nanosheets

**DOI:** 10.3390/ma10111282

**Published:** 2017-11-08

**Authors:** Duan Zhang, Tanhua Jia, Ran Dong, Dengyun Chen

**Affiliations:** 1 School of Physics, Beijing Institute of Technology, Beijing 100081, China; jiatanhua0223@163.com (T.J.); dongran528@icoud.com (R.D.); chendengyun130@163.com (D.C.); 2 Beijing Key Laboratory for Nano-Photonics and Nano-Structure, Elementary Educational College, Capital Normal University, Beijing 100048, China

**Keywords:** GaSe, photoluminescence, strain, temperature dependence

## Abstract

Two-dimensional A^III^B^VI^ layered semiconductors have recently attracted great attention due to their potential applications in piezo-phototronics and optoelectronics. Here, we report the temperature-dependent photoluminescence (PL) of strained and unstrained GaSe flakes. It is found that, as the temperature increases, the PL from both the strained (wrinkled) and unstrained (flat) positions show a prominent red-shift to low energies. However, for the flat case, the slope of PL energy versus temperature at the range of 163–283 K is about −0.36 meV/K, which is smaller than that of the wrinkled one (−0.5 meV/K). This is because more strain can be introduced at the freestanding wrinkled position during the temperature increase, thus accelerates the main PL peak (peak I, direct band gap transition) shift to lower energy. Additionally, for the wrinkled sheet, three new exciton states (peaks III, IV, and V) appear at the red side of peak I, and the emission intensity is highly dependent on the temperature variation. These peaks can be attributed to the bound exciton recombination. These findings demonstrate an interesting route for optical band gap tuning of the layered GaSe sheet, which are important for future optoelectronic device design.

## 1. Introduction

Two-dimensional (2D) layered crystals such as graphene have been intensively investigated in the past decade due to their attractive mechanical, electrical, optical, and chemical properties that are absent in the corresponding bulk counterparts [1,2,3,4,5]. Compared with the semimetallic graphene (zero band gap) and insulating hexagonal boron, the most recently investigated A^III^B^VI^ layered semiconductors are especially interesting for electronic and optoelectronic applications because of their nonzero band gap and strong photoluminescence in single-layer form [6,7,8,9,10]. Gallium selenide (GaSe) is a representative layered A^III^B^VI^ binary chalcogenides, and is an intriguing semiconductor with an indirect band gap of 2.11 eV and a direct band gap of only 25 meV higher [11,12,13]. It demonstrates interesting electrical and optical properties, such as a high on/off ratio [7,14], large anisotropic Hall mobility [7,15], good gas sensibility [16], and strong second-harmonic generation [17]. More recently, it has also been shown that the electronic band structures and PL intensity of layered GaSe can be effectively tuned via the elastic strain engineering [18,19,20]. These flexible electrical and optical properties make it an appealing candidate for mechanically compliant optoelectronics with applications in piezo-phototronics, optoelectronics, wearable devices and human–machine interfaces [21,22,23,24]. So far, many efforts have been made to investigate the strain-dependent electrical and optical properties of GaSe nanosheets, however, as a critical parameter in optoelectronic devices design and fabrication, the thermal effects on the electrical and optical properties are less known, especially for the strained GaSe nanosheets.

In this work, we prepare the GaSe nanosheets onto a flexible polydimethylsiloxane (PDMS) substrate by mechanical exfoliation. GaSe wrinkles are then created by bending the PDMS substrate. Furthermore, we perform the temperature-dependent PL measurements on the flat (unstrained) and wrinkled (strained) GaSe positions. It is found that, the main PL peak (peak I, direct band gap transition) of wrinkled GaSe sheet redshifts from 2.04 eV to 1.98 eV, as the temperature increases from 163 K to 293 K, which is more sensitive than that of flat GaSe sheet. This can be understood by the fact that more strain can be induced at the free-standing wrinkled GaSe position during the temperature increase. Additionally, at the wrinkled position, three new exciton states (peaks III, IV, and V) can be observed. These peaks are highly dependent on the temperature increase and gradually disappear at about 163 K. These peaks can be attributed to the bound exciton recombination. Our findings demonstrate an interesting route for optical band gap tuning of the layered GaSe sheet, which are important for future optoelectronic device design.

## 2. Results and Discussion

**Figure 1:**
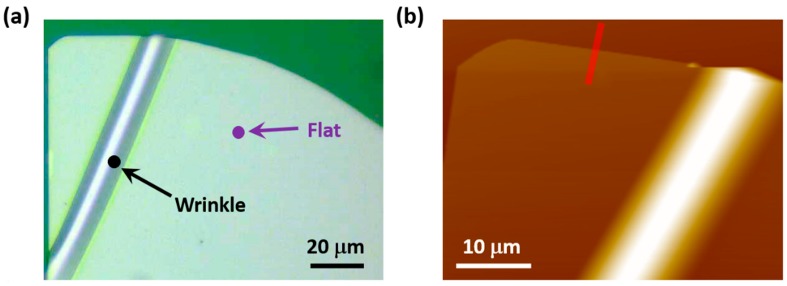

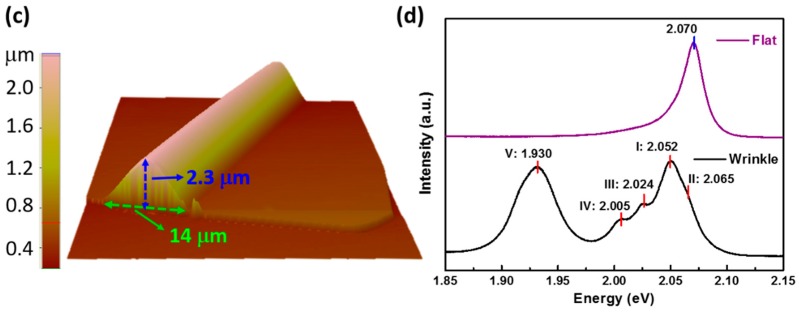
PL spectra from flat and wrinkled positions measured at 100 K. (**a**) Optical image of the obtained GaSe flake; (**b**) AFM image of GaSe flake; (**c**) three-dimensional AFM image of GaSe wrinkle structure; (**d**) PL spectra measured at 100 K, where the detecting positions are marked in (**a**). The peak positions are obtained by Lorenz–Gaussian fitting.

Figure 1a shows the optical image of the obtained GaSe flake on PDMS substrate. By the bending process (see Materials and Methods for details), a prominent wrinkle can be observed. The atomic force microscopy (AFM) measurements (Figure 1b,c) demonstrate that the flat GaSe flake has the thickness about 200 nm and the wrinkled structure is 14 μm in diameter and 2.3 μm in height. The line intensity profiles for the AFM image at flat and wrinkled positions are shown in Figure S3 in supplementary materials. Owing to the wrinkle, a certain value of localized elastic strain can be generated in the flake, which can be evaluated by the equation: ε = τ/ρ, where τ is the half-thickness of the GaSe sheet and ρ is the radius of curvature at a specific point. We assume that the central layer within the GaSe stack remains unstrained, while the outer curved surface of the sheet is stretched by ε and the inner curved surface is compressed by −ε. Details of the strain calculation can be found in the Supplementary Materials. In Figure 1d, the PL spectra measured at 100 K from flat (top) and wrinkled (bottom) points are shown. At low temperature (100 K), the main PL peak at 2.07 eV can be observed from the flat position. For the wrinkled one (ε ~ 0.5%, as calculated in the Supplementary Materials), the strain-induced funnel effect on the electronic band structure makes the major PL peak split into two peaks [24]. One strong peak is located at ~2.052 eV (peak I, direct band gap transition). The other one (peak II, indirect band gap transition) is located at 2.065 eV, which is much weaker than peak I and presented as a shoulder. Besides the splitting of the main PL peak, several new peaks can also be observed at lower energies, such as peaks III, IV, and V located at 2.024 eV, 2.005 eV, and 1.93 eV, respectively.

**Figure 2:**
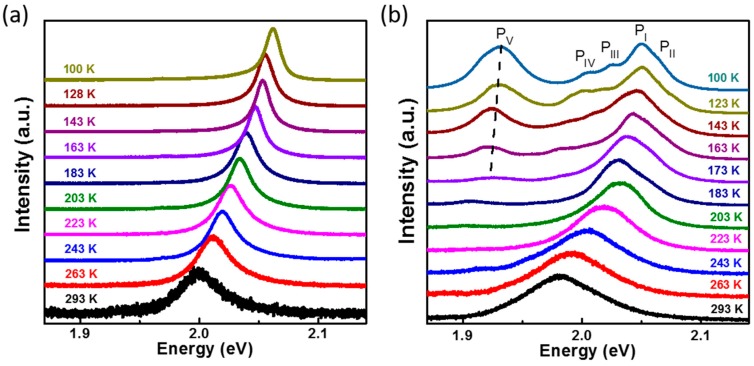
Temperature-dependent PL spectra of flat (unstrained) and wrinkled (strained) GaSe in the range of 100 to 293 K: (**a**) Flat GaSe; (**b**) Wrinkled GaSe.

**Figure 3:**
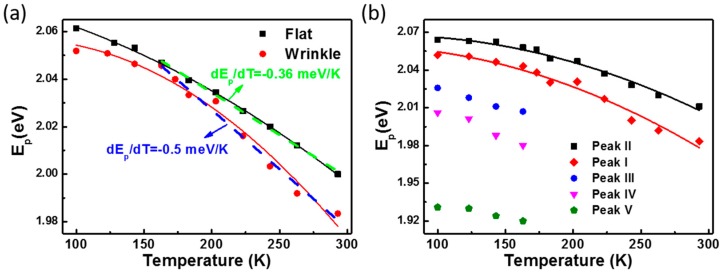
The temperature-dependent evolution of PL peaks from flat (unstrained) and wrinkled (strained) positions. (**a**) Black squares: the major PL emission from flat position. Red dots: peak I from the wrinkled position. The solid curves are the least-squares fits of data with the Varshni empirical equation. The dashed lines are the linear fits; (**b**) The five PL peaks from the wrinkled position.

To understand the thermal effects on the electrical and optical properties of the GaSe flake, temperature-dependent PL measurements are further performed on the flat and wrinkled positions, respectively. Figure 2 presents the temperature-dependent evolution of PL spectra of the GaSe. Each spectrum has been shifted vertically for clarity. For the flat (unstrained) case in Figure 2a, it is found that the energy of PL emission redshifts from 2.06 eV to 1.99 eV, as the temperature increases from 100 K to 293 K. At the same time, the full width at half maximum (FWHM) of PL peaks change from 13 meV to 42 meV. This can result from the enhanced electron–phonon coupling during the temperature increase [25,26]. However, for the wrinkled (strained) one, besides the redshift of peaks I and II, peaks III, IV, and V become weaker and gradually disappear when the temperature increases. Figure 3a summarizes the evolution of the PL emission from flat position and the main PL peak (peak I) from the wrinkled position as a function of temperature. Both the flat and wrinkled GaSe flakes demonstrate a prominent decrease in the optical band gap during the increase of temperature, which can result from the increased electron–phonon interactions and lattice expansion at high temperatures [25,26]. The experimental temperature dependence of the PL emission energy can be well fitted by the empirical Varshni equation [25], E_p_(T) = [E_g_(0) − E_x_] − (αT^2^)/(T + β), where the E_g_(0) is the energy band gap at 0 K, E_x_ is the exciton binding energy, and α and β are the Varshni coefficients, as described by the dark (flat) and red (wrinkle) curves in Figure 3a. We find that the emission energy difference between strained and unstrained GaSe gradually becomes larger as the temperature increases from 163 K to 283 K. So we choose this specific temperature range to investigate the strain and temperature influences on the energy band gap of GaSe nanosheet. Interestingly, at this high-temperature region (higher than 163 K), the temperature dependence of the PL peak can be fitted by a simpler linear function, E_p_(T) = [E_g_(0) − E_x_] − αT, as described by the dashed lines. For the flat position, the slope of E_p_(T) versus temperature at the range of 163–283 K is about −0.36 meV/K, which is smaller than that of the wrinkled one (−0.5 meV/K). This can be due to the extra strain introduced by the expansion of GaSe lattice during the increase of temperature; especially at the freestanding wrinkled position, the thermal expansion can bring more strain to the GaSe flake. This can be confirmed by the evolution of peaks I and II at the wrinkled position. As shown in Figure 3b, the energy difference between these two peaks increased from 13 meV to 28 meV when the temperature increased from 100 K to 293 K. We have previously reported that, owing to the strain-induced funnel effect on the electronic band structure of the GaSe sheet, the PL peak will split into two peaks, where the major peak shifts to lower energy and a new exciton state emerges as a weak shoulder at the high energy side of the major PL peak [24]. As the strain increases, the energy difference between these two peaks becomes larger. Here, for the wrinkled case, besides the major PL peak I at 2.052 eV, a weak shoulder at ~2.065 eV (peak II) can also be observed from the PL spectrum measured at 100 K, as shown in Figure 1d. Furthermore, when the temperature increases, the shoulder becomes more and more prominent, such as the PL spectra at 163 K, 173 K, and 183 K in Figure 2b. The intensity ratio of P I to P II is also shown in Figure S5. It is found that the ratio decreases gradually with the increase of temperature from 100 to 293 K, which also indicates that the thermal expansion can introduce strain to the wrinkled GaSe sheet, thus changing the relative quantum efficiencies of direct band gap transition (peak I) and indirect band gap transition (peak II). Based on these facts, we can confidently deduce that the thermal expansion will introduce more strain to the freestanding wrinkled GaSe sheet, which accelerates the shift of major PL peak I to lower energy during the increase of temperature. For the flat position, the GaSe sheet and PDMS substrate can expand simultaneously during the temperature increase, which greatly offsets the strain. So the temperature variation can induce less strain on the flat position, and the PL spectrum from the flat position at 293 K demonstrates a single emission peak. A thinner GaSe sheet (~80 nm in thickness) with the wrinkled structure (2 μm in height and 20 μm in diameter) was also fabricated, as shown in Figure S6. We undertook temperature-dependent PL measurements at different locations on the wrinkled structure. The results demonstrate that, the top of the wrinkle shows a larger slope value (the dependence of PL emission energy on temperature) than that measured at the middle and flat positions, which is consistent with our discussion above.

**Figure 4:**
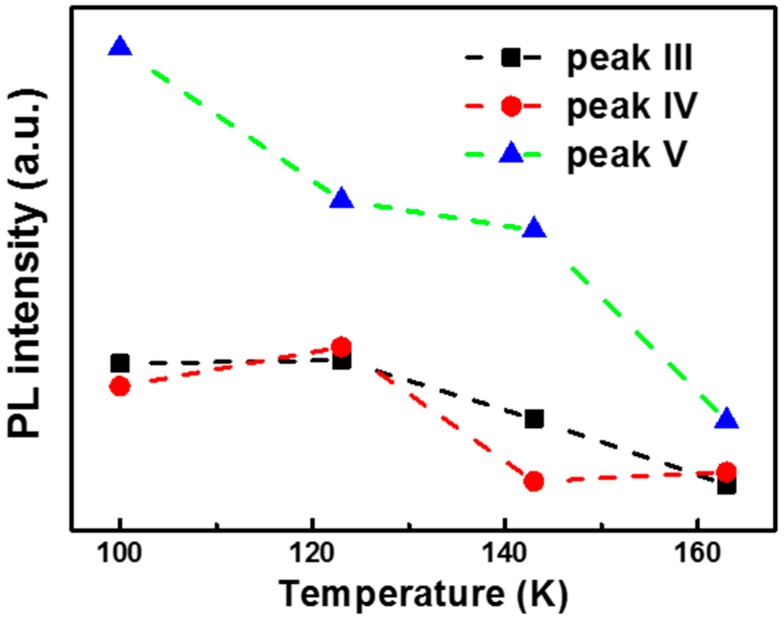
Temperature-dependent PL intensity of peaks III, IV, and V for the strained GaSe flake.

Additionally, for the wrinkled position, the emission intensity of peaks III, IV, and V gradually decreases and finally disappears at a temperature about 163 K, as shown in Figure 4. By comparing with the flat case, we can find that two conditions are necessary to make these peaks observable: low temperature and strain. Because the emission intensity of these peaks is highly dependent on the temperature, these peaks can be ascribed to the bound exciton recombination. When the temperature increases, more and more free excitons are released from the bound state, which makes the emission gradually disappear, as observed in the PL spectra in Figure 2b.

## 3. Materials and Methods

Few-layer GaSe flakes were transferred onto a flexible PDMS substrate by mechanical exfoliation from bulk 2H-GaSe purchased from HQ Graphene (Groningen, The Netherlands) using GEL film (Gel-Pak), similar to that for graphene [1]. To improve the adhesion of GaSe on PDMS, a layer of SU8 photoresist (~3 μm) was spin-coated onto the PDMS substrate in advance. The thickness of the obtained GaSe nanosheets was identified by combining the optical contrast in a microscope and AFM measurements. To introduce localized strain to the obtained GaSe nanosheets, the flexible PDMS were mechanically bent, as illustrated in Figure S1. This bending process leads to compression of the PDMS/SU8 top side surface and stretching of the under-surface. The relatively low ductility and excellent bend ability of GaSe allow wrinkles to be generated on the GaSe sheets. After releasing the flexible substrate, the substrate recovers its relaxed position, but strained wrinkles on the GaSe sheets remain. The direction of generated wrinkles are mainly dependent on the press direction. As shown in Figure S2, the PDMS substrate was pressed along the “x” direction, and the generated wrinkles were approximately parallel to the “y” direction. The PL spectroscopy measurements were performed on a confocal Raman spectrometer (Bruker, Karlsruhe, Germany) at an excitation of 532 nm. The excitation power is 5 mW with an integration time of 1 s. For the temperature-dependent measurements, a Linkam TST 350 liquid nitrogen low-temperature stage was mounted onto the micro-Raman system. The accuracy of the temperature control was ±1 K.

## 4. Conclusions

In summary, the wrinkled GaSe nanosheet are simply fabricated by bending the flexible PDMS substrate. The temperature-dependent PL measurements show that, the main PL peak (peak I, direct band gap) from the wrinkled position is more sensitive to the temperature variation than that from flat position. Especially, at the range of 163–283 K, the slope of PL energy versus temperature of wrinkled one is about 0.5 meV/K which is larger than that of flat one (0.36 meV/K). Besides the faster red shift of direct band gap energy, the indirect band gap transition (peak II) also become stronger gradually as the temperature increases. These results indicate that more strain can be introduced at the freestanding wrinkled position during the temperature increase, thus tuning the band gap structure of GaSe sheet. Additionally, three new exciton states (peaks III, IV, and V) are observed at the red side of the main PL peak I, and the emission intensity is highly dependent on the temperature variation. These peaks can be assigned to the bound exciton recombination. These findings is beneficial to the understanding of GaSe electrical and optical properties and demonstrate an interesting route for band gap tuning of the layered GaSe sheet, which are important for future optoelectronic device design.

## Supplementary Materials

The following materials are available online at www.mdpi.com/1996-1944/10/11/1282/s1. Figure S1: Schematic of the process to fabricate GaSe wrinkles; Figure S2: The optical image of the wrinkled GaSe sheet; Figure S3: The line intensity profile of the AFM image; Figure S4: Calculation of strain within GaSe wrinkles; Figure S5: The temperature dependence of the intensity ratio of P I (direct band gap transition) to P II (indirect band gap transition); Figure S6: Temperature-dependent PL measurements at different locations of the wrinkled region.
