# Supplementary material for: Temperature-Dependent Photoluminescence Emission from Unstrained and Strained GaSe Nanosheets
Source: Materials (Basel). 2017 Nov 8;10(11):1282. doi: 10.3390/ma10111282 (PMC5706229; doi:10.3390/ma10111282)
Supplement: Supplementary file 1 [file materials-10-01282-s001.pdf]

Supplementary Materials: Temperature-Dependent Photoluminescence Emission from Unstrained and Strained GaSe Nanosheets

Duan Zhang, Tanhua Jia, Ran Dong, and Dengyun Chen

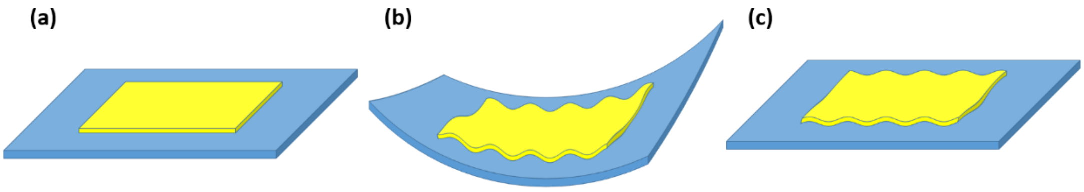

Figure S1. Schematic of the process to fabricate GaSe wrinkles. (**a**) Mechanically exfoliated GaSe flakes are transferred onto the PDMS substrate; (**b**) the PDMS substrate is bent to create wrinkles; (**c**) PDMS substrate is released and GaSe wrinkles remain on the substrate.

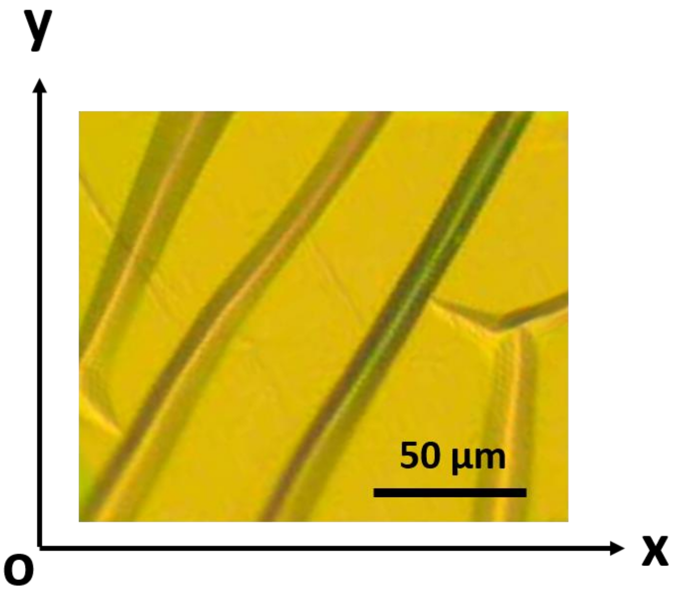

Figure S2. The optical image of the wrinkled GaSe sheet. The wrinkle direction is mainly dependent on the press direction. The PDMS substrate was pressed along the "x" direction, and the generated wrinkle structures were approximately parallel to the "y" direction.

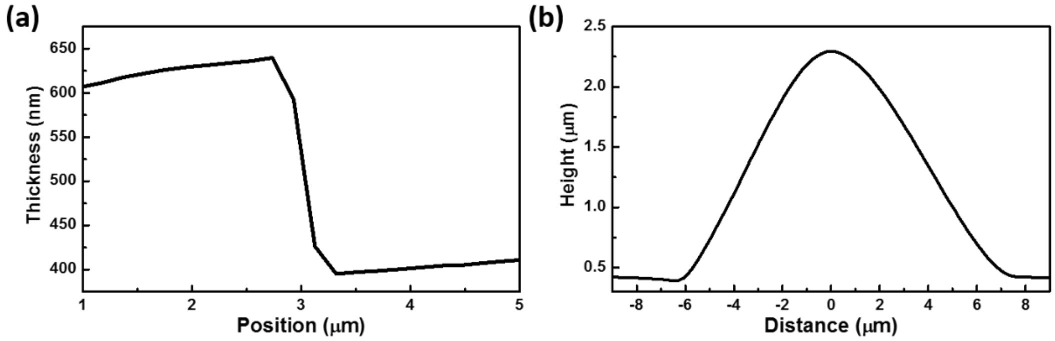

Figure S3. The line intensity profile of the AFM images. (**a**) Flat position; (**b**) Wrinkled position.

Strain Calculation

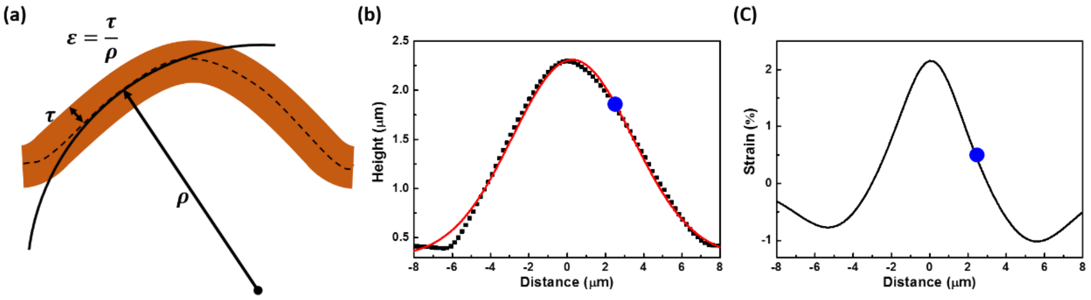

Figure S4. Calculation of strain within GaSe wrinkles. The blue spot indicates the detecting position on the wrinkled GaSe sheet with the strain at about 0.5%.

When a GaSe nano sheet is wrinkled, as shown in Figure S3a, the value of the strain at a specific point can be derived by the relationship:

$$\varepsilon = \frac{\tau}{\rho},$$

where τ is the half-thickness of the GaSe sheet and ρ is the radius of curvature at the point. We assume that the central layer within the GaSe stack remains unstrained, while the outer curved surface of the sheet is stretched by ε and the inner curved surface is compressed by -ε, as demonstrated in Figure S4a. To determine the radius of curvature ρ, the height profile of the AFM image is first fitted with a Gaussian peak function G(x), as illustrated in Figure S4b. Since the thickness of the GaSe sheet is much smaller than the dimensions of the radius of curvature of the wrinkle, the radius of the outer surface can be considered to be the radius of curvature of the central layer. Therefore the radius of the curvature can be calculated as follows:

$$\rho(x) = \frac{\left[1 + G'(x)^2\right]^{\frac{3}{2}}}{G''(x)}.$$

Thus the corresponding strain mapping of the upper outer surface of the wrinkle in the GaSe sheet is shown in Figure S4c, where the positive values represent tensile strain and the negative values mean compressive strain. We find that the peak of the wrinkle experiences the maximum tensile strain, while there are transition zones between flat and wrinkled regions that experience compressive strain.

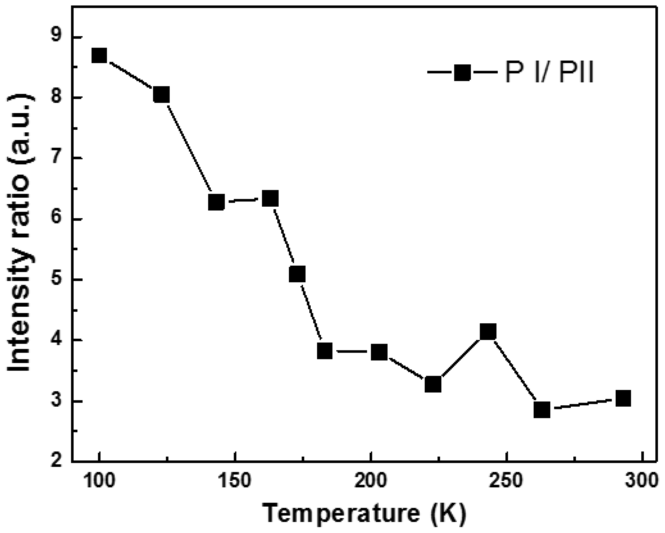

Figure S5. The temperature dependence of intensity ratio of P I (direct band gap transition) to P II (indirect band gap transition).

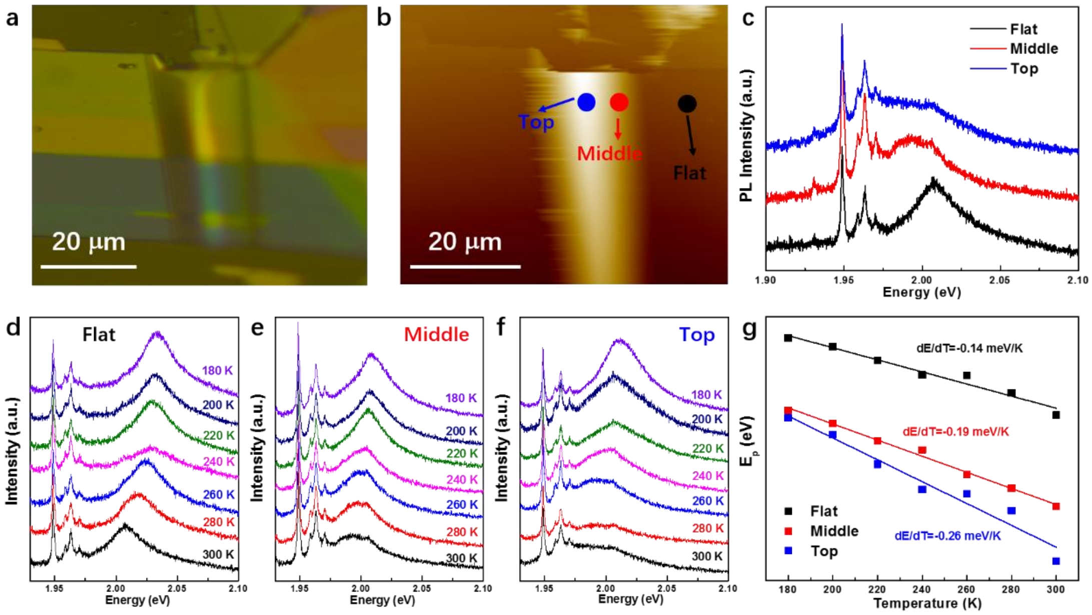

Figure S6. The temperature-dependent PL measurements at different locations of the wrinkled region. (**a**) Optical image of a wrinkled GaSe sheet; (**b**) AFM topography of the wrinkled GaSe sheet (~80 nm in thickness) with the wrinkle structure 2 μm in height and 20 μm in diameter; (**c**) room-temperature PL spectra measured from flat, middle, and top positions, as indicated in the AFM image; (**d–f**) temperature-dependent PL measurements at the flat, middle, and top positions; (**g**) temperature dependence of P I emission energy measured from flat, middle, and top positions.
